# Scalable Electronic Ratchet with Over 10% Rectification Efficiency

**DOI:** 10.1002/advs.201902428

**Published:** 2019-12-13

**Authors:** Olof Andersson, Joris Maas, Gerwin Gelinck, Martijn Kemerink

**Affiliations:** ^1^ Complex Materials and Devices Department of Physics Chemistry and Biology (IFM) Linköping University SE‐581 83 Linköping Sweden; ^2^ Holst Centre/TNO High Tech Campus 31 5656 AE Eindhoven The Netherlands; ^3^ Molecular Materials and Nanosystems Department of Applied Physics Eindhoven University of Technology 5600 MB Eindhoven The Netherlands

**Keywords:** field effect transistors, indium–gallium–zinc oxide (IGZO), modeling, ratchets, rectification

## Abstract

Electronic ratchets use a periodic potential with broken inversion symmetry to rectify undirected (electromagnetic, EM) forces and can in principle be a complement to conventional diode‐based designs. Unfortunately, ratchet devices reported to date have low or undetermined power conversion efficiencies, hampering applicability. Combining experiments and numerical modeling, field‐effect transistor‐based ratchets are investigated in which the driving signal is coupled into the accumulation layer via interdigitated finger electrodes that are capacitively coupled to the field effect transistor channel region. The output current–voltage curves of these ratchets can have a fill factor >> 0.25 which is highly favorable for the power output. Experimentally, a maximum power conversion efficiency well over 10% at 5 MHz, which is the highest reported value for an electronic ratchet, is determined. Device simulations indicate this number can be increased further by increasing the device asymmetry. A scaling analysis shows that the frequency range of optimal performance can be scaled to the THz regime, and possibly beyond, while adhering to technologically realistic parameters. Concomitantly, the power output density increases from ≈4 W m^−2^ to ≈1 MW m^−2^. Hence, this type of ratchet device can rectify high‐frequency EM fields at reasonable efficiencies, potentially paving the way for actual use as energy harvester.

## Graphical abstract

This study investigates ratchets driven by two sets of asymmetrically spaced interdigitated finger electrodes situated within a dielectric layer on top of an indium–gallium–zinc oxide field‐effect transistor. The maximum experimentally observed efficiency is above 10% at 5 MHz. Via simulations, engineering guidelines are established for increasing the power output and efficiency into the THz range and beyond.

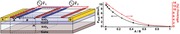

## 1 Introduction

A ratchet, in the scientific sense, is a device that is characterized by a periodic potential that lacks inversion symmetry. Through this characteristic it may rectify nondirectional forces, i.e., periodic forces or noise signals that accumulate to zero when integrated over time, that drive the ratchet out of equilibrium. These broad criteria leave the design of a ratchet rather open and consequently diverse types have been presented in literature.^1^, ^2^

The ratchet concept was popularized through Feynman's treatment of Smoluchovski's thought experiment with the ratchet and pawl presented in his lecture series more than 50 years ago.^3^ Regarding practical applications, only few devices have been presented so far, mostly with the general function to sort particles of different sizes or types.^4^, ^5^, ^6^, ^7^, ^8^, ^9^, ^10^, ^11^, ^12^ Microscopically, polystyrene beads of different sizes in a fluid can be sorted in asymmetric landscapes of either mechanical,^8^, ^11^, ^12^ magnetic,^7^, ^9^ or electrostatic nature.^4^, ^10^ Macroscopically, mixtures of granular matter of different sizes can be sorted horizontally by laterally shaking a container with sawtooth‐patterned walls.^13^ random motions of motile cells can be directed by an asymmetric Christmas tree ratchet structure.^14^, ^15^

Different kinds of electronic ratchets have been studied in literature.^16^, ^17^, ^18^, ^19^, ^20^ Many of these are variants of the so‐called on‐off ratchet that use an asymmetric sawtooth‐shaped electrostatic potential to confine charge carriers in local potential minima in the on‐state, while in the off‐state the electrostatic potential is removed so that the charge carriers can spread. In the subsequent on‐state the charge carriers will again be confined in the local minima of the sawtooth potential and, due to the asymmetry, a net movement of charge carriers in one direction can be achieved.^19^, ^20^ In general, the output power density and efficiency of these devices is low, precluding any practical use. Alternatively, Mikhnenko et al. reported a maximum power efficiency of 6% in the kHz regime and <2% in the MHz regime for an organic ionic ratchet, based on a transistor structure where the charge carriers are driven by a time‐varying gate voltage and rectified by unequal injection/extraction‐rates at the source and drain contacts due to imbalanced ion concentrations achieved by a prior bias stress.^21^ Although this device lacks periodicity and basically rectifies as an ionic Schottky diode, it represents the currently highest documented power conversion efficiency of rectification.

To actually use a ratchet for energy harvesting would require it to take some abundant energy source such as electromagnetic (EM) radiation as input. Although visible light is readily harvested by solar cells based on the photovoltaic effect, these devices are limited by equilibrium thermodynamics as reflected in the Shockley–Queisser (SQ) limit. As a consequence, they are unsuited to harvest the IR fraction of the EM spectrum. Theoretically it has been shown that ratchets can reach Carnot efficiency,^22^ and as they fundamentally operate as nonequilibrium devices, they are not bound by the SQ limit; in a recent paper it was argued that a bottom‐up designed ratchet, based on the bulk photovoltaic effect in a ferroelectric material, surpassed the SQ limit.^23^ Although this work has been disputed, the principle remains valid.^24^, ^25^ A top‐down, lithographically defined ratchet acting as an energy harvester was presented by Pan et al. who investigated a thermal ratchet consisting of a spiral antenna in series with a self‐switching nanodiode reaching a power conversion efficiency of 0.02%.^26^

Here, we experimentally demonstrate a scalable electronic ratchet that reaches a power output density ≈6 W m^−2^ at a power conversion efficiency exceeding 10% at 5 MHz driving frequency. The ratchet is based on a modified field effect transistor (FET) and is fabricated by standard photolithography. Using numerical device simulations, we discuss the scaling of power efficiency and power density with material and device parameters—notably charge carrier mobility and feature size—and argue that, without loss of efficiency, power densities in the MW m^−2^ range at THz frequencies and beyond are feasible using technologically reasonable parameters.

## 2 Results

**Figure 1:**
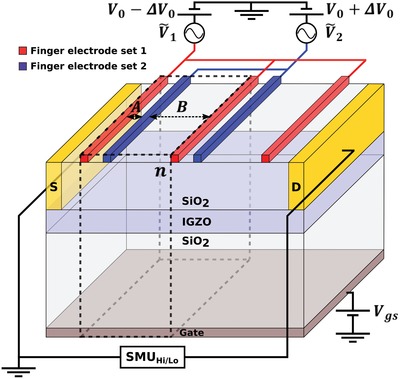
Schematic of the investigated FET ratchet device. In addition of the bottom‐gate top‐contact transistor configuration there is a second layer of the gate dielectric material, SiO_2_, on top of the semiconductor (Indium Gallium Zinc Oxide, IGZO) with half the thickness of the gate dielectric layer. On top of the second layer of gate dielectric material sits two sets of asymmetrically spaced finger electrodes (red/blue bars). Dashed lines indicate the repeat unit; the full device consists of *n* = 16 repeat units plus one extra finger of finger electrode set 1 that terminates the device. The horizontal distance from the source and drain contacts to the closest finger electrode is 5 µm, the interelectrode spacings are *A* = 4 µm and *B* = 16 µm, the electrode width is 2 µm, the channel width is 60 µm.

The architecture of the ratchet devices investigated in this study is shown in **Figure** **1**. It is based on a top‐contact bottom‐gate FET.^27^ The gate electrode was formed by patterning a 100 nm molybdenum‐chromium (MoCr) metal layer, followed by deposition of 200 nm SiO_2_ layer by plasma enhanced chemical vapor deposition (PECVD) as the gate insulator. Indium–gallium–zinc oxide (IGZO) was used as active channel material, and was deposited by RF‐sputtering, to a layer thickness of 24 nm. Apart from being a very stable compound, IGZO was chosen since its mobility in combination with the length scales of our devices leads to efficiency maxima that conveniently sit just below the upper frequency limit of our equipment. On top of the semiconductor 100 nm PECVD SiO_2_ was deposited as etch‐stop‐layer. Contact openings for the source and drain of the transistors were etched with a CF_4_ chemistry in a reactive ion etch process. The devices were finalized by MoCr deposition and patterning of the source/drain metal contacts and the finger electrodes set1 and set2. All process steps are standard flat panel display industrial fabrication processes. The substrate was 320 × 352 mm glass. Prior to all measurements it was checked that there were no significant leakage currents between the (source, drain) contacts and the gate, nor between the two sets of finger electrodes, nor between the finger electrodes and the contacts

The device is driven out of equilibrium via sinusoidal voltages, ${\tilde{V}}_{1,2}=V_{\text{Ampl}.}\;\cdot \text{sin}(2\pi ft+\phi_{1,2})$ with amplitude *V*_Ampl._, frequency *f* and adjustable phase difference ϕ_*i*_, being applied to the two sets of finger electrodes. The phase angle of finger electrode set 1 ϕ_1_ is fixed to 0°, hence ϕ_2_ equals the phase difference between set 1 and 2 and will be referred to as ϕ. All voltages are applied with respect to the source, hence *V*_GS_ sets the background charge carrier concentration in the IGZO channel. The field‐effect mobility of electrons in our devices was calculated in the linear region from measured transfer curves to be typically µ_lin._ ≈ 25 cm V^−1^ s^−1^ as indicated in Figure S1 (Supporting Information). To suppress threshold voltage shifts usually observed in IGZO when exposing devices to prolonged gate biases, all measurements were carried out under Úrk conditions.^28^, ^29^ Typical threshold voltage shifts in our devices during measurements amount to around 1 V and are corrected for by taking transfer curves before and after measurements. All measurements are performed under (dark) ambient conditions at room temperature inside a Janis probe station.

To complement our experimental results, we developed a simple numerical simulation model. The channel is modeled as a 1D chain of cells and the model only considers the drift part of the full drift‐diffusion equation, $\bar{j_{n}}=\;qn\mu_{n}\bar{E}$, where $\bar{j_{n}}$ is the electron current density, *q* is the elementary electric charge, *n* is the electron density, µ_*n*_ is the electron mobility, and $\bar{E}$ is the electric field. Charge conservation is implemented via the continuity equation, $\bar{\nabla }\cdot \;\bar{j_{n}}=\;-q\partial_{t}n$, where ∂_t_ indicates the time derivative. Making the gradual channel approximation allows replacing Poisson's equation by a local capacitive coupling. Each grid cell *i* of the channel has a specific (areal) capacitance, *C_i_*, that couples it to the nearest gate voltage, *V*_*g*,*i*_, that can be either the constant back gate voltage or the time‐varying voltage on one of the two sets of finger electrodes, such that electron density and local potential are coupled via (*V_i_*(*t*) − *V*_*G*,*i*_(*t*)) = *n_i_*(*t*)/*C_i_*, where *n_i_*(*t*) is the areal charge density at grid cell *i*. The drift and continuity equations are solved by forward integration in time with a suitably chosen time step. Further details can be found in Figure S2 (Supporting Information), but we should stress that all model parameters are independently determined, i.e., no fitting has been done. In Figure S3 (Supporting Information), we compare simulations from a full 2D drift‐diffusion model with the 1D drift‐only model for a single repeat unit with periodic boundary conditions (i.e., no contacts). For the latter model, also a full device simulation (with contacts) is added to the comparison. Although quantitative differences between the simulations are present, the predicted trends are similar. Since full device simulations are needed to reproduce experimentally measured fill factors (vide infra) and calculation times of full devices with the 2D drift‐diffusion model are prohibitively long, the 1D drift‐only model is used throughout the rest of this work.

In principle, ratchets are nonequilibrium devices that are characterized by a periodic potential that lacks inversion symmetry. However, it can be rather nonintuitive which driving conditions are required to achieve a net output. The inversion symmetry in our ratchet devices can be broken in different ways. First, by the phase difference, ϕ, between the time‐varying potentials on the asymmetrically spaced finger electrodes. The additional terminating finger electrode in electrode set 1 further breaks the symmetry, although numerical simulations (see paragraph below) showed that this has only a limited effect on the device behavior. Third, applying a nonzero offset difference, ± Δ*V*_0_, will introduce a DC background sawtooth potential on top of the oscillation. The drive with nonzero Δ*V*_0_ was named forward drive in Ref. 19 and complicates both operation and analysis by introducing additional requirements on the amplitude‐offset ratio. Although this is not pursued here, Δ*V*_0_ = 0 is also more compatible with a practical rectifying device in which the finger electrodes are (driven by) actual antennas. Furthermore, in Figure S4 (Supporting Information), we demonstrate that the output at symmetric drive (Δ*V*_0_ = 0) is very similar in magnitude as in forward drive while having a simpler frequency dependence. Specifically, maximum and minimum output occur at ϕ ≈ 90°, 270°, and ϕ ≈ 0°, 180°, respectively, irrespective of drive frequency. Hence, the simpler symmetric driving scheme is chosen for the purpose of this study. Unless stated otherwise, the base device regarded is (as described in Figure 1) *A* = 4 µm, *B* = 16 µm, with 16 repeat units and an extra terminating finger of finger electrode set 1, with a driving scheme where the two sets of finger electrodes have the same voltage offset, *V*_0_, and are driven by sinusoidal voltages with an amplitude 2.5 V at given phase difference at *V*_GS_ − *V*_th_ = 10 V.

Figure S5 (Supporting Information) shows that for symmetric drive the measured device output is (practically) zero at ϕ = 0° and ϕ = 180°. Intuitively, this can be understood from the very minor effect of the additional terminating finger electrode. Ignoring this electrode altogether, it can easily be visualized that charges are either symmetrically shuttled in and out of the channel (at ϕ = 0°) or symmetrically shuttled back and forth between the two pairs of finger electrodes (at ϕ = 180°) over the span of a full oscillation and no net current is established. Note that the underlying absence of asymmetry at these driving phase angles is independent of the (ratio of the) short and long finger spacings *A* and *B*; as mentioned above, the extra finger electrode of set 1 only causes a slight inequivalence in the different charge transport directions in the channel at ϕ = 0° and ϕ = 180°. Consequently, the optimal phase differences for symmetric drive are close to ϕ = 90° and ϕ = 270°, which give (virtually) equal currents in opposite directions.

**Figure 2:**
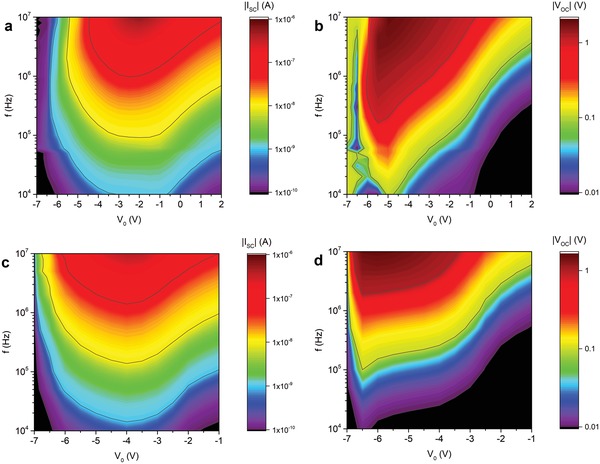
Contour plots of the time‐averaged (DC) *I*_SC_ and *V*_OC_ versus offset *V*_0_ and drive frequency *f* for a,b) measurements and c,d) simulations. *V*_GS_ − *V*_th_ = 10 V, *V*_Ampl._ = 2.5 V, ϕ = 270°.

**Figure** **2**a displays the measured DC device current under short circuit conditions, *I*_SC_, versus offset bias *V*_0_ and drive frequency for a phase difference ϕ = 270°. The overall shape shows a current that increases with frequency and drops steeply at large negative *V*_0_ and more gradually toward large positive *V*_0_. The roll‐off at high negative *V*_0_ is a direct consequence of channel depletion below the finger electrodes, blocking any DC current in the channel. In contrast, at high positive *V*_0_ the mean local charge carrier density will be very high and effectively overwhelm the density variation in the channel induced by the oscillating finger electrodes and no net current will be extracted. Hence, in between, at moderate negative offset values the optimal offset bias with respect to *I*_SC_ is obtained.

The monotonic increase in DC current with drive frequency is a direct consequence of the net charge that is displaced per oscillation cycle being roughly constant in this frequency regime. For the current mobilities, the frequency regime where the charge motion can no longer keep up with the oscillating field and the displaced charge per cycle starts to show nonmonotonic behavior lies above 10^8^ Hz, which is beyond our measurement capabilities, see Figure S4c (Supporting Information). The measured monotonic increase in *I*_sc_ is in stark contrast with the behavior measured before for similar devices in forward drive with a low‐mobility organic semiconductor as active layer, where current reversals could be observed well below 10^6^ Hz.^19^ The reasons for the difference are the different driving scheme, c.f., Figure S4c,d (Supporting Information) and discussion above, and the 3 orders of magnitude mobility difference that shifts the current reversals to beyond our maximum measurement frequencies, c.f., Figure S4c (Supporting Information) and the discussion below on scaling. Below we will also show that maximal efficiencies are reached at frequencies well below the high frequency roll‐off, and this regime will not be further pursued.

Previously, it was assumed that there exists a linear relation between the short circuit current *I*_SC_ and the open circuit voltage *V*_OC_ of this type of electronic ratchets, in line with what is commonly observed for ratchets.^21^, ^30^, ^31^ In case the slope of the *I–V* curve is set by the total channel resistance, which only weakly depends on offset *V*_0_,^30^ contour plots of *V*_OC_ should strongly resemble those of *I*_SC_. Figure 2b shows this is not the case. Compared to *I*_SC_ we acquire the optimal offset for *V*_OC_ at more negative values of *V*_0_, close to where channel pinch‐off occurs.

Numerically simulated *I*_SC_ and *V*_OC_ contour plots are shown in Figure 2c,d. Despite the simplicity of the model, they reproduce the experimental trends in *I*_SC_ and *V*_OC_ well, both regarding magnitude and shape; the shift in offset space is due to a nonzero threshold voltage in the experiment and will be present in all plots over offset. Note that these simulations do not contain any freely adjustable parameters since geometries are known from fabrication and a measured electron mobility is used.

**Figure 3:**
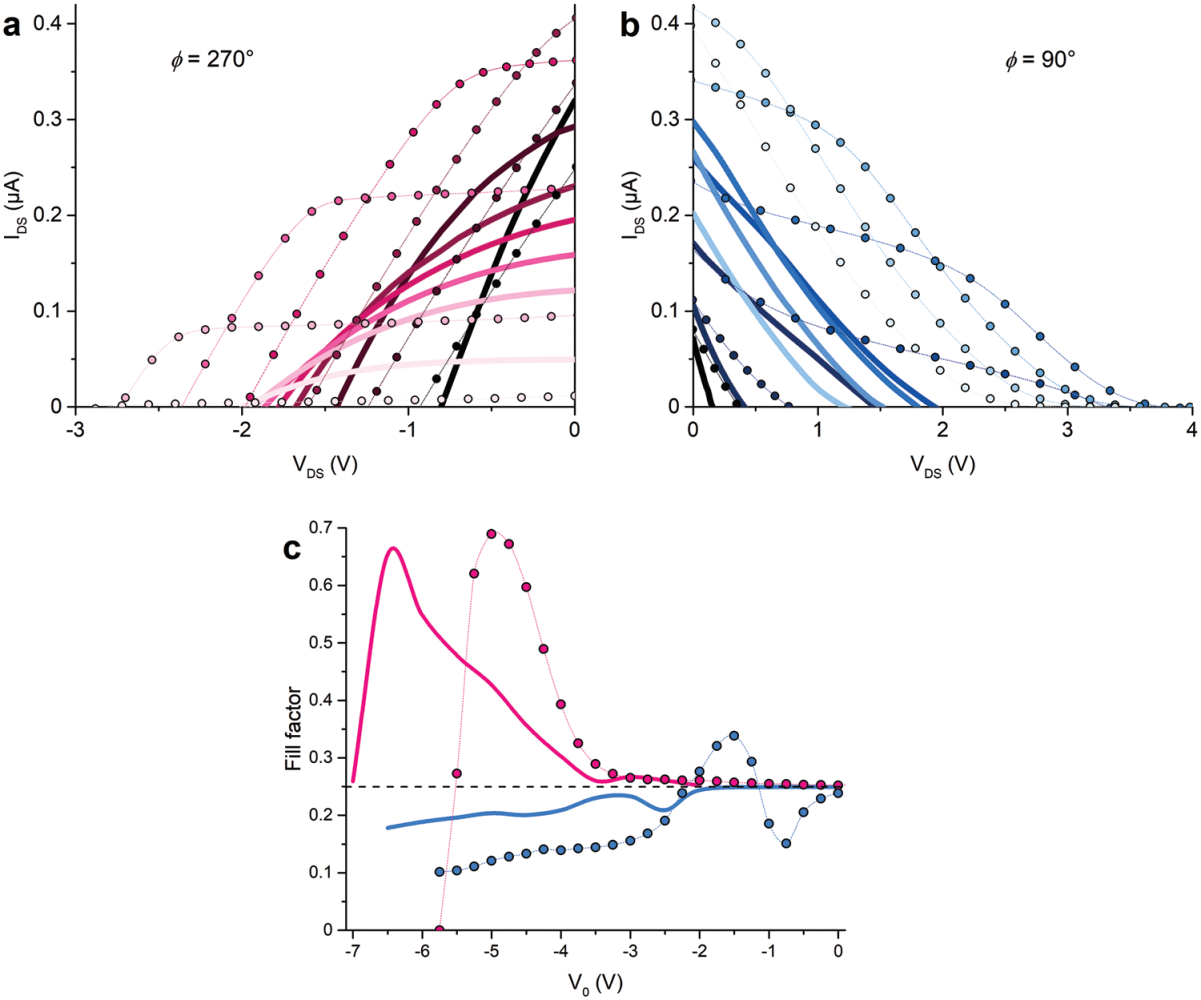
Measured (symbols) and simulated (full lines) *I–V* curves for ϕ = 270° a), and ϕ = 90° b). a) Measurements for ϕ = 270° show *I–V* curves for *V*_0_ from −2.5 to −5.5 V in −0.5 V steps (dark to light colors) and simulations for *V*_0_ from −3.5 to −6.5 V in −0.5 V steps. b) Measurements for ϕ = 90° show *I*–*V* curves (|*I*_DS_| for comparison reason) for *V*_0_ from 0 to −3 V in −0.5 V steps and simulations for *V*_0_ = −2 to −5 V in −0.5 V steps. Insets show the fill factor versus offset. *V*_GS_ − *V*_th_ = 10 V, *V*_Ampl._ = 2.5 V.

**Figure** **3**a,b displays *I–V* curves at different offsets *V*_0_, showing that the device produces a net output power in the second and fourth quadrants for ϕ = 270° (a), and ϕ = 90°, respectively—note −*I*_DS_ is plotted for ϕ = 90°. Clearly, the ratchet *I–V* curves bear strong similarity to those of solar cells and hence we use the corresponding nomenclature of short circuit current *I*_SC_, open circuit voltage *V*_OC_, and fill factor (FF) throughout the manuscript. It is important, though, that in solar cells the semiconductor bandgap plays a crucial role in determining the amount of absorbed energy (light), setting *I*_SC_, and in determining the resulting Fermi level splitting, setting *V*_OC_. In our ratchet devices the semiconductor bandgap is of no significance as excitation occurs via the capacitive coupling between the finger electrodes and the channel and does not lead to interband transitions as in photovoltaic cells.

A distinct shape difference is seen between the *I–V* curves for phase differences of 270° and 90°, both in measurements and simulations. This appears to conflict with the (near) equivalence of driving the device at ϕ = 270° and ϕ = 90°, as the extra set 1 finger electrode near the drain was argued to only generate a minute difference. Instead, the disparity occurs due to *V*_DS_ for ϕ = 270° being swept from 0 V to negative values to counteract the positive source–drain current, while for ϕ = 90° *V*_DS_ must be swept from 0 V to positive values to counteract the negative *I*_SD_. This leads to different potentials and accordingly charge density landscapes in the channel as positive *V*_GS_ increases accumulation in the channel, whereas negative *V*_GS_ depletes the channel. Simultaneous reversal of source and drain contacts would therefore lead to a (near) equivalence of ϕ = 90° and ϕ = 270°, which is not particularly interesting, and we chose to investigate both “depleting” and “accumulating” output characteristics. Throughout this paper we will simply refer to ϕ = 90° and ϕ = 270° and by implication refer to the associated sign of any applied *V*_DS_.

For ϕ = 270° the *I–V* curves are linear at more positive offset values but, in contrast to other reported ratchets, remarkably convex for more negative offsets.^21^, ^30^, ^31^ Consequently, the fill factors are much higher than 1/4, almost reaching 0.7 as shown in the inset. For ϕ = 90° we see a more concave shape instead, especially at *V*_DS_ closer to *V*_OC_, and thus mostly obtain fill factors below 1/4. Although fill factors > 1/4 are acquired for ϕ = 90° over a limited *V*_0_ range, they never reaching the highest values of ϕ = 270°. Intuitively, one may therefore expect the highest power conversion efficiency of the 270° case to exceed that of the 90° case, and to sit at more negative offsets. Below, we shall demonstrate that this is indeed the case.

The simulations in Figure 3 reproduce the main trends in the measurements in that ϕ = 270° yields convex *I–V* curves which have fill factor > ¼, whereas ϕ = 90° yields concave *I–V* shapes with fill factors <1/4. The small offset in *V*_0_ range used in simulations and experiments reflects a nonzero threshold voltage of the finger gates, as is also visible in Figure 2. The nonlinear *I–V* curves only occurs in simulations when contacts are included. The maximum output voltages and currents are lower in simulations than measurements. These differences are attributed to the 1D of the simulated model and, especially, the neglect of diffusion, which may be significant in this frequency range as we see that both the output voltage and current are higher in the 2D drift‐diffusion model, as shown in Figure S3 (Supporting Information). Taking 5 µm as a typical length scale of our devices, the frequency at which diffusion becomes relevant can be estimated by *f* ≈ *D*/Δ*x* ^2^ = µ*k*_B_ *T*/Δ*x* ^2^ ≈ 2.5 MHz for a mobility *µ* = 25 cm^2^ V^−1^ s^−1^.

**Figure 4:**
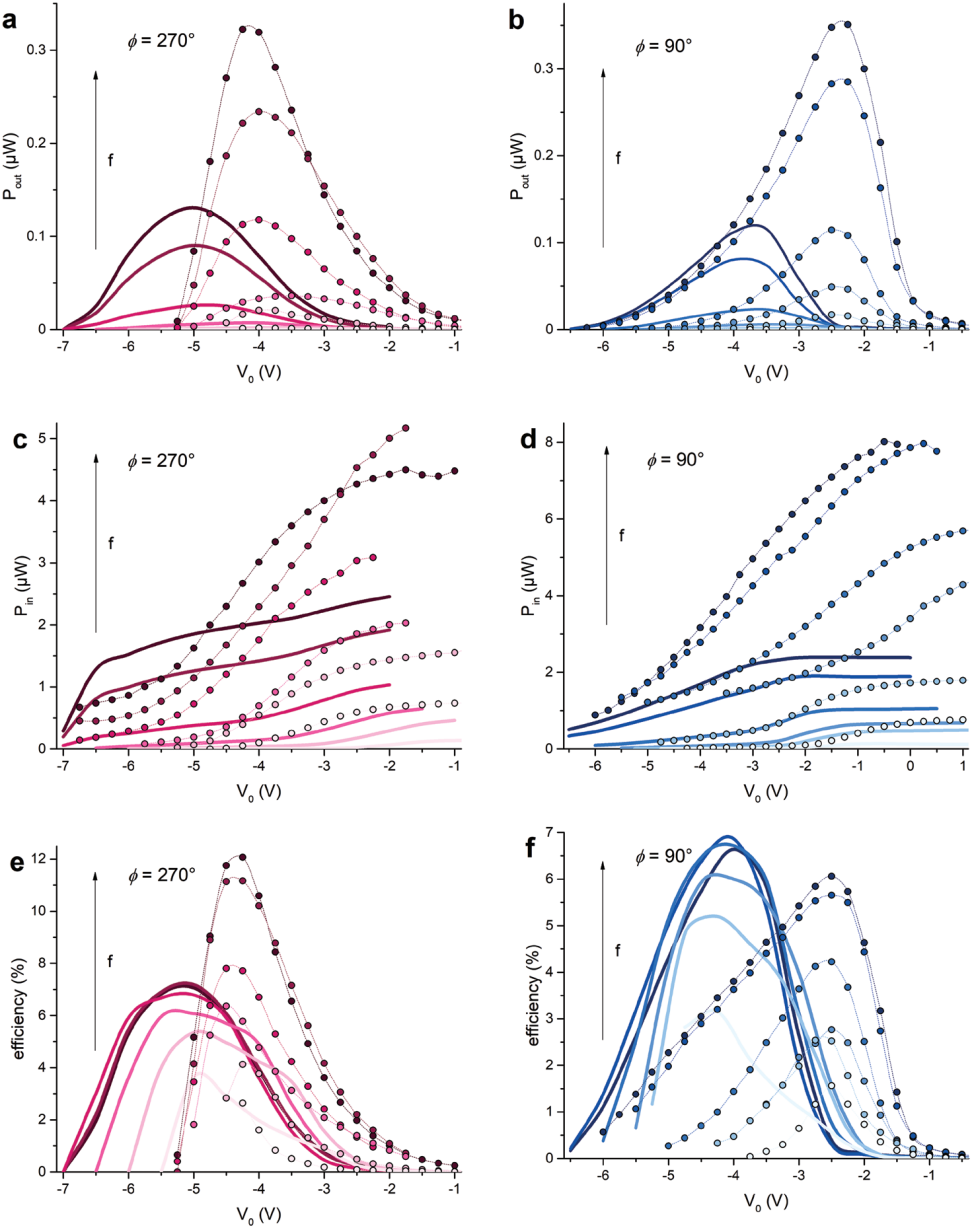
Measurements (symbols with lines) and simulations (full lines) for 100, 500, 1, 2, 4, and 5 MHz from light to darker color, ϕ = 270° (a,c,e) and ϕ = 90° (b,d,f). a,b) power output at maximum power point from *I*–*V* curves versus offset, c,d) power input, $P_{\text{in}}=\frac{P_{\text{in},\text{SC}}+P_{\text{in},\text{OC}}}{2}$, versus offset, e,f) efficiency, $\eta \;=\frac{P_{\text{out}}}{P_{\text{in}}}\;$, versus offset. Measurements and simulations have for some frequencies been slightly shifted in offset‐space to have peak maxima of *P*_out_ sit at the same offset‐value at each frequency for comparison reasons. *V*_gs_ − *V*_th_ = 10 V, *V*_Ampl._ = 2.5 V.

From measured and simulated *I–V* curves the power output at maximum power point is extracted and plotted versus offset bias in **Figure** **4**a,b. In line with the observations in Figure 3, the simulations reproduce the shape but underestimate the peak value by a factor 2–3. While the power supplied to the system via the interdigitated finger electrodes, and thereby the power conversion efficiency, are easily obtained in simulations, this is far from trivial in the actual experiment. The reason for this is the presence of significant background impedances in the system that are associated with (lossy) capacitive couplings to the environment and the impossibility to accurately measure the complex currents flowing to each set of finger electrodes.

To determine the input power, *P*_in_, in the real device, the signal output of an impedance analyzer is used to drive one set of the finger electrodes with AC signal ${\tilde{V}}_{\text{AC},\text{IA}}$. A function generator drives the other set with the desired phase difference, where the AC signal is described by ${\tilde{V}}_{\text{AC},\text{IA}}e^{i\phi }$. The impedance analyzer signal input is connected to the shunted source and drain contacts in short‐circuit mode and to the drain in open‐circuit mode; in the latter case, the source is left floating. This setup yields the *total* complex impedance of the system from the perspective of the set of finger electrodes that is driven by the impedance analyzer, and, since we know the driving voltage and phase difference of the other set of finger electrodes, also from the perspective of the other set of finger electrodes. From this we need to derive the power dissipation caused by the individual sets of finger electrodes for each value of *V*_0_.

**Figure 5:**
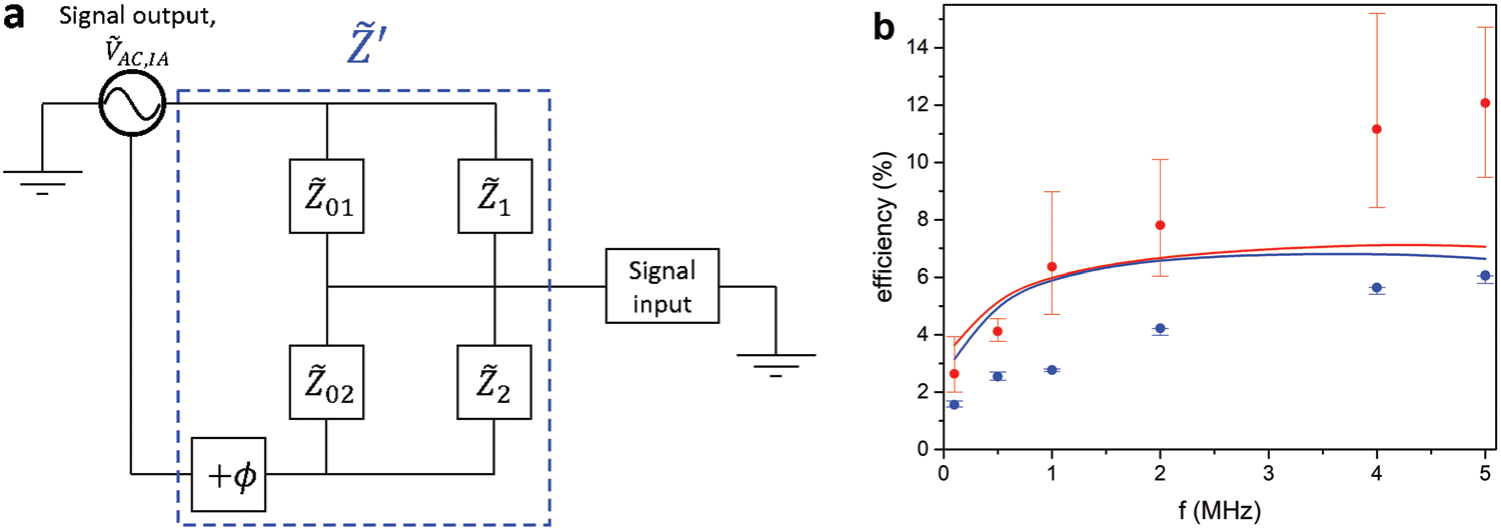
a) Circuit diagram of the device. The complex impedances ${\tilde{Z}}_{01}$ and ${\tilde{Z}}_{02}$ represent stray capacitances and lossy transmission lines to each respective set of finger electrodes, while ${\tilde{Z}}_{1}$ and ${\tilde{Z}}_{2}$ correspond to the complex impedance of the coupling of each set of finger electrodes to the accumulation layer in the transistor channel. The time‐varying voltage signal from the output of the function generator is equivalently expressed as a phase‐shifted (+ϕ) signal output from the impedance analyzer. b) Maximal power conversion efficiency versus frequency. Measurements (symbols with error bars) and simulations (full lines) for ϕ = 270° (red) and ϕ = 90° (blue). Symbols in measurements correspond to $P_{\text{in}}=\frac{P_{\text{in},\text{SC}}+P_{\text{in},\text{OC}}}{2}$, and the upper and lower error bar correspond to *P*_in_ weighted with respect to the current at maximum power point, *I*_MPP_,: $P_{\text{in},\text{OC}}+\frac{I_{\text{MPP}}}{I_{\text{SC}}}(P_{\text{in},\text{SC}}-P_{\text{in},\text{OC}})$ and *P*_in_ weighted with respect to the voltage at maximum power point, *V*_MPP_,: $P_{\text{in},\text{SC}}-\frac{V_{\text{MPP}}}{V_{\text{SC}}}(P_{\text{in},\text{SC}}-P_{\text{in},\text{OC}})$, where MPP is the maximum power point.

To achieve this, we model the two sets of finger electrodes as parallel circuit elements, each with an absolute impedance value, $\left|{\tilde{Z}}_{j}\right|$, and phase angle, θ_*j*_, (*j* = 1, 2) that both can depend on *V*_0_ and accompanying discussion. Each ${\tilde{Z}}_{j}$ is connected in parallel to a background impedance ${\tilde{Z}}_{0,j}$ containing stray capacitances and cabling. The components that dissipate power are modeled in the circuit diagram in **Figure** **5**a. Since there is only a phase difference between the input signal from the impedance analyzer and the function generator we can simplify the circuit by adjusting the concerned impedances (${\tilde{Z}}_{02}$ and ${\tilde{Z}}_{2}$) for this phase difference and model the whole system as being driven by the impedance analyzer. The resulting total impedance, $\tilde{Z}\prime$, is actually what is measured with the impedance analyzer and is given by $\tilde{Z}\prime =1/\left(\left(\frac{{\tilde{Z}}_{01}+{\tilde{Z}}_{1}}{{\tilde{Z}}_{01}{\tilde{Z}}_{1}}\right)+\left(\frac{{\tilde{Z}}_{02}+{\tilde{Z}}_{2}}{{\tilde{Z}}_{02}{\tilde{Z}}_{2}}\right)\cdot \frac{1}{e^{i(-\phi )}}\right)$.

The background impedances ${\tilde{Z}}_{01}$ and ${\tilde{Z}}_{02}$ can be measured sequentially by depleting the channel ($\left|{\tilde{Z}}_{1}\right|\left|{\tilde{Z}}_{2}\right|,\to \infty$) and grounding one set of finger electrodes at a time, see Figure S7 (Supporting Information). Even with the background dissipation removed, determining the input power requires extracting 4 parameters (magnitude and phase angle of the current to each set of finger electrodes) from two knowns (magnitude and phase angle of the *total* output current). It turns out that in this particular case this is well possible by first approximating $\left|{\tilde{Z}}_{2}\right|$ in terms of $\left|{\tilde{Z}}_{1}\right|$ as $\left|{\tilde{Z}}_{2}\right|\;\text{=}\frac{17}{16}\;\cdot \left|{\tilde{Z}}_{1}\right|$ via the relation $\frac{\left|{\tilde{Z}}_{1}\right|}{\left|{\tilde{Z}}_{2}\right|}=\frac{\#\;\text{of}\;\text{set}\;1\;\text{finger}\;\text{electrodes}}{\#\;\text{of}\;\text{set}\;2\;\text{finger}\;\text{electrodes}}\;=\frac{16}{17}\;$ due to the capacitive nature of the coupling of the finger electrodes to the channel, and subsequent fitting of $\left|{\tilde{Z}}_{1}\right|$, θ_1_, and θ_2_ to the measured impedance. Further details of the method are given in Figure S8 (Supporting Information).

Unfortunately, the methodology sketched above does not allow measuring impedance at other than short‐ and open‐circuit conditions. In particular, the voltage at maximum power point, *V*_MPP_, cannot be applied without introducing additional unknowns to the system. Therefore, we approximate the experimental input power at maximum power point in three ways: a) as the average of *P*_in_ at short‐circuit and open‐circuit conditions $\frac{P_{\text{in},\text{SC}}+P_{\text{in},\text{OC}}}{2}$, b) weighted with respect to current at maximum power point, *I*_MPP_,: $P_{\text{in},\text{OC}}+\frac{I_{\text{MPP}}}{I_{\text{SC}}}(P_{\text{in},\text{SC}}-P_{\text{in},\text{OC}})$, and c) weighted with respect to voltage at maximum power point, *V*_MPP_,: $P_{\text{in},\text{SC}}-\frac{V_{\text{MPP}}}{V_{\text{SC}}}(P_{\text{in},\text{SC}}-P_{\text{in},\text{OC}})$. Note that these three approximations for *P*_in_ are equal for linear *I–V* curves (fill factor = 1/4). The input power in simulations is, in contrast, determined at maximum power point by applying the corresponding *V*_DS_ and is calculated by multiplying field and current in each grid cell and summarizing over the whole channel for each time step and integrating over time.

Figure 4c,d compares measurements and simulations of *P*_in_. Generally the simulated *P*_in_ is lower than the measured *P*_in_ but overall, the correspondence is reasonable in shape and magnitude and both display an S‐shape with a finite, roughly constant asymptote toward positive offset‐values, and a zero asymptote toward larger negative offset values where the finger electrodes start depleting the channel. As for the output power, the simulations underestimate the experiments by a factor ≈2–4. Consequently, the experimental power conversion efficiency η = *P*_out_/*P*_in_ is rather well reproduced by the simulations; for ϕ = 270° experimental efficiencies are higher than simulations, mostly due to the higher fill factor, while for ϕ = 90° measurements give slightly lower efficiencies, see Figure 4e,f. The flipped triangular shapes for ϕ = 270° e) and ϕ = 90° f) are well captured by the model. As was explained above, the difference between ϕ = 270° and ϕ = 90° results from the opposite polarity of the source–drain bias at maximum power point and can therefore only be reproduced by a full device model and not by a model of a perfectly periodic (infinite) system.

To the best of our knowledge, the data in Figure 4 are the highest experimentally determined power conversion efficiencies of rectification by an electronic ratchet. A summary of the measured and simulated power efficiencies of our devices is given in Figure 5b. The relatively large error bar for ϕ = 270° is due to the large (≫1/4) fill factor that causes a significant dependence of the input power on the choice of weighing *P*_in_ on *P*_in,SC_ and *P*_in,OC_, as explained above and in the caption. The error originating from the threshold voltage shifts during the measurements is depicted in Figure S9 (Supporting Information) and does not affect any of our conclusions. Importantly, both experiments and simulations prove that FET‐based ratchets can rectify at relevant efficiencies over a broad frequency range. Although this is not further pursued here due to experimental constraints, simulations in Figure S10 (Supporting Information) show that further efficiency increase by almost a factor 2 should be possible for larger driving amplitudes. As detailed in Figure S10 (Supporting Information) the reason is that while the output power at MPP increases quadratically with increasing amplitude, due to both *V*_MPP_ and *I*_MPP_ being linear in *V*_Ampl._, the input power is more weakly dependent on *V*_Ampl._.

**Figure 6:**
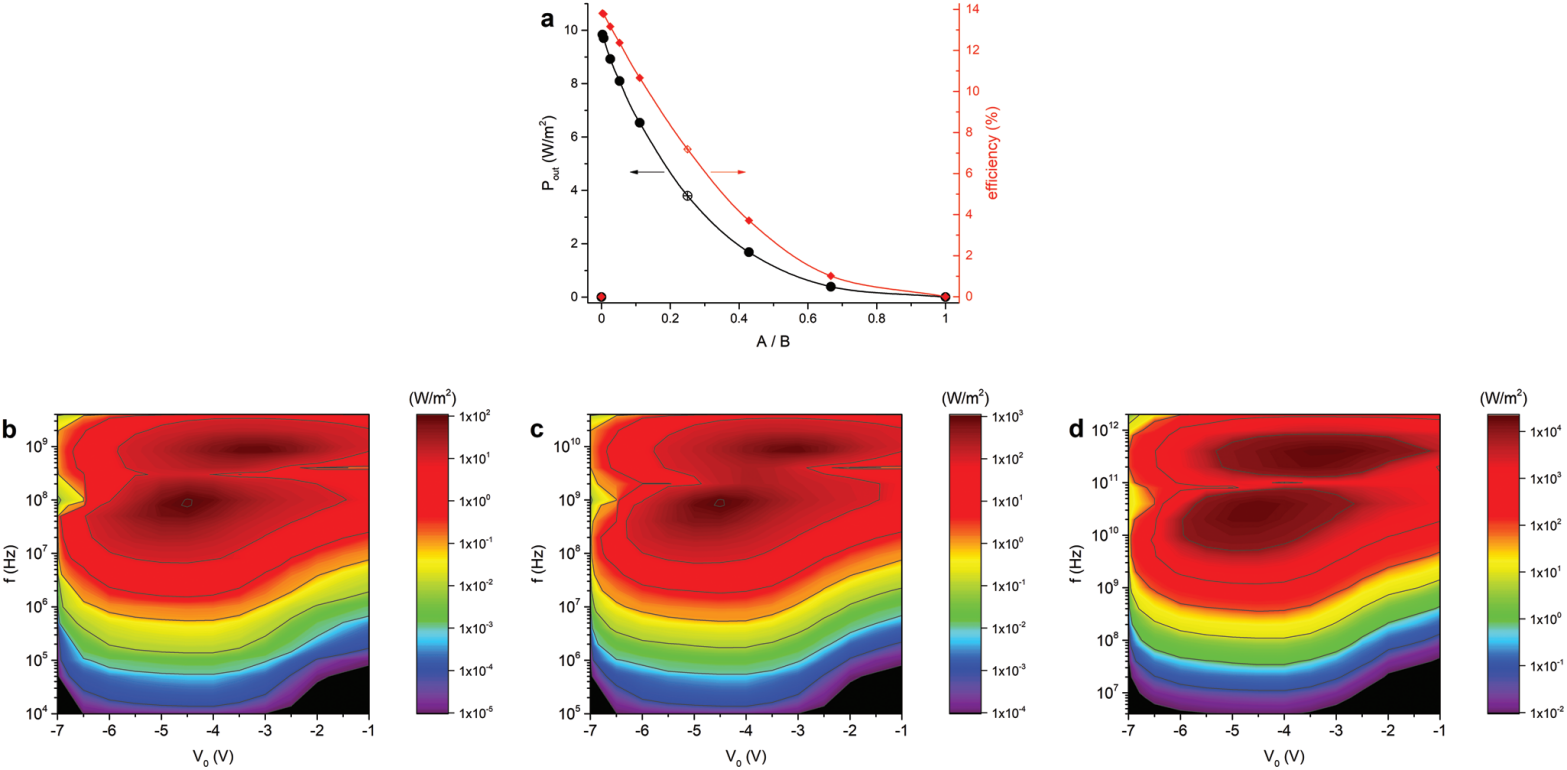
a) Power output density (at maximum efficiency, 6 MHz) and maximum efficiency versus asymmetry *A*/*B* for *A*+*B* = 20 µm. The measured base device has *A* = 4 µm, *B* = 16 µm (crossed symbol corresponding at *A*/*B* = 0.25). b–d) Power output density simulations versus offset and frequency for the base device (b), 10 × higher µ (c) and 20 × decreased horizontal length scales (d). Note that the power density is calculated using the channel area, and that a current reversal occurs with increasing frequency after the first maximum, c.f., Figure S4c, Supporting Information).

The efficiency measurements were limited in frequency space by the 5 MHz bandwidth of the impedance analyzer. Even though the maximum output power maxima are located at much higher frequencies, ≈80 and ≈800 MHz according to simulations in **Figure** **6**b, the maximum efficiency is located close to 5 MHz as supported by simulations that stretch to higher frequencies (Figure S11, Supporting Information). Hence, we do not expect any significant increase in efficiency at higher frequencies than measured herein. However, both output power and efficiency are markedly dependent on the asymmetry which in this system can be altered by varying the ratio between the short and long distances *A* and *B* between the finger electrodes, c.f., Figure 1. We simulated the system varying *A* and *B* while keeping the horizontal length of one period *n* fixed at 24 µm (*A+B* = 20 µm, finger electrode width 2 µm), i.e., the same value as our physical device. Figure 6a shows that one may expect an efficiency increase by roughly a factor 2 when moving from the actual device to *A* = 0.0625 µm and *B* = 19.9375 µm, which is still realistic from a fabrication perspective. Decreasing the shorter length, *A*, further toward the nanoscale might introduce quantum effects, and because these are not treated in the used drift‐diffusion formalism, we do not include simulations in this length scale. Interestingly, the output power density at point of maximum efficiency increases more than a factor 2 from 3.8 to 9.8 W m^−2^, using the channel area for normalization while ignoring additional areas for, e.g., secondary electronics or antennas. Note that in the limiting case *A/B* → 0, the finger electrodes from the two sets will be in contact and will no longer constitute a ratchet, while at *A/B* = 1 we are no longer breaking the periodic spatial asymmetry. Note also that the absolute numbers for a real device might be higher than this as we have seen above that the output power and efficiency can be higher in measurements than in simulations. Moreover, the data in Figure 6a have been obtained for a constant (low) driving amplitude of 2.5 V, c.f., Figure S10 (Supporting Information).

For potential application of ratchets as high‐frequency rectification devices, it is imperative that the high efficiency persists beyond the MHz regime studied experimentally herein. To this end, we have investigated the scalability of this type of ratchet device by varying relevant parameters in simulations. Comparing Figure 6b,c shows that upon increasing the mobility by a factor 10 to *µ* = 250 cm^2^ V^−1^ s^−1^, the output power spectrum remains invariant apart from a linear shift in frequency space; simultaneously the maximum output power density increases linearly by a factor 10. Likewise, the output power spectrum of a device with all horizontal length scales decreased by a factor 20 shifts quadratically to higher frequencies and accordingly higher output powers, see Figure 6d. The smallest feature of the simulated device is the 100 nm width of the finger electrodes. For both devices in Figure 6c,d the efficiency spectrum only shifts in frequency space compared to base device, leaving the maximum efficiency practically constant (Figure S12, Supporting Information).

The scaling of the 2D power and efficiency spectra with µL^−2^ discussed above is consistent with that found by Roeling et al. for the frequency at maximum current under forward drive.^32^ The important practical implication is that with technologically realistic parameters over 10% rectification efficiency at and beyond THz frequencies should be well‐accessible using state‐of‐the‐art lithography and high‐mobility transistors. Although the simple device layout and the driving scheme described above cannot one‐on‐one be transferred to these frequencies, there is no fundamental problem in designing conventional, or even plasmonic antennas that effectively pick up EM radiation and couple it (phase‐shifted) into the channel at these and even higher frequencies. Likewise, the drift formalism used in simulations can be expected to function reliably till the (reciprocal) charge carrier scattering time, which is in the sub‐ps (above‐THz) regime for high‐electron‐mobility transistors, HEMT's, with mobilities in the 1000 cm^2^ V^−1^ s^−1^ range and above.^33^ Assuming furthermore a 10 nm feature size, as is currently available through electron beam lithography and in the near future through extreme ultraviolet lithography,^34^, ^35^ allows scaling down our base device by a factor 200 to *A*_2_ = 20 nm, *B*_2_ = 80 nm and a finger electrode width of 10 nm. The same oscillating scheme used throughout the paper then yields a (simulated) maximum efficiency (of 6.6%) at ≈8 THz and a corresponding power output density of 1.2 MW m^−2^; the absolute maxima in output power density sit at even higher frequencies (≈10^14^ Hz (NIR) and ≈10^15^ Hz (UV) for the parameters used). Even with the limitations noted, it is inspiring that direct rectification of (far‐ or near‐IR) light should be possible with suitably designed ratchets—something that so far has proven elusive with conventional rectennas.^36^

In conclusion, we have investigated an FET‐based ratchet driven by sinusoidal voltage on two sets of interdigitated finger electrodes inside the channel area. We found that this type of ratchet device exhibits nonlinear *I–V* curves in the parameter range where maximum output and efficiency occur, with a maximum fill factor close to 0.7. Via impedance measurements the input power was determined and subsequently a maximum power conversion efficiency of over 10% was achieved at 5 MHz. We developed a 1D drift‐only simulation model to support our measurements and investigate higher frequencies than accessible by our equipment. Using the model, we investigated the parameter dependency and found that with accessible technology it should be possible to shift the maximum power conversion efficiency to at least the THz regime. The maximum power efficiency can be further increased by increasing the device asymmetry, i.e., by minimizing the ratio between the short and long distances between neighboring finger electrodes. Following these guidelines one can engineer a ratchet device to be a highly efficient rectifier for a desired frequency range, possibly competing with current rectenna technology which, although reaching efficiency levels well over 50%, operates at relatively low frequencies in the GHz range.^37^, ^38^

## Conflict of Interest

The authors declare no conflict of interest.

## Supporting information

Supporting Information
