# Supplementary material for: Scalable Electronic Ratchet with Over 10% Rectification Efficiency
Source: Adv Sci (Weinh). 2019 Dec 13;7(3):1902428. doi: 10.1002/advs.201902428 (PMC7001629; doi:10.1002/advs.201902428)
Supplement: Supplementary file 1 — Supporting Information [file ADVS-7-1902428-s001.pdf]

Copyright WILEY-VCH Verlag GmbH & Co. KGaA, 69469 Weinheim, Germany, 2019.

ADVANCED SCIENCE
Open Access

Supporting Information

for *Adv. Sci.,* DOI: 10.1002/advs.201902428

Scalable Electronic Ratchet with Over 10% Rectification Efficiency

*Olof Andersson, Joris Maas, Gerwin Gelinck, and Martijn Kemerink**

Supporting Information to:

Scalable electronic ratchet with over 10% rectification efficiency

Olof Andersson[1], Joris Maas[2], Gerwin Gelinck[2,3], Martijn Kemerink[1]

[1] Complex Materials and Devices, Dept. of Physics, Chemistry and Biology (IFM), Linköping University, SE-581 83 Linköping, Sweden.

[2] Holst Centre/TNO, High Tech Campus 31, 5656 AE, Eindhoven, The Netherlands.

[3] Molecular Materials and Nanosystems, Dept. of Appl. Physics, Eindhoven University of Technology, 5600 MB Eindhoven, The Netherlands.

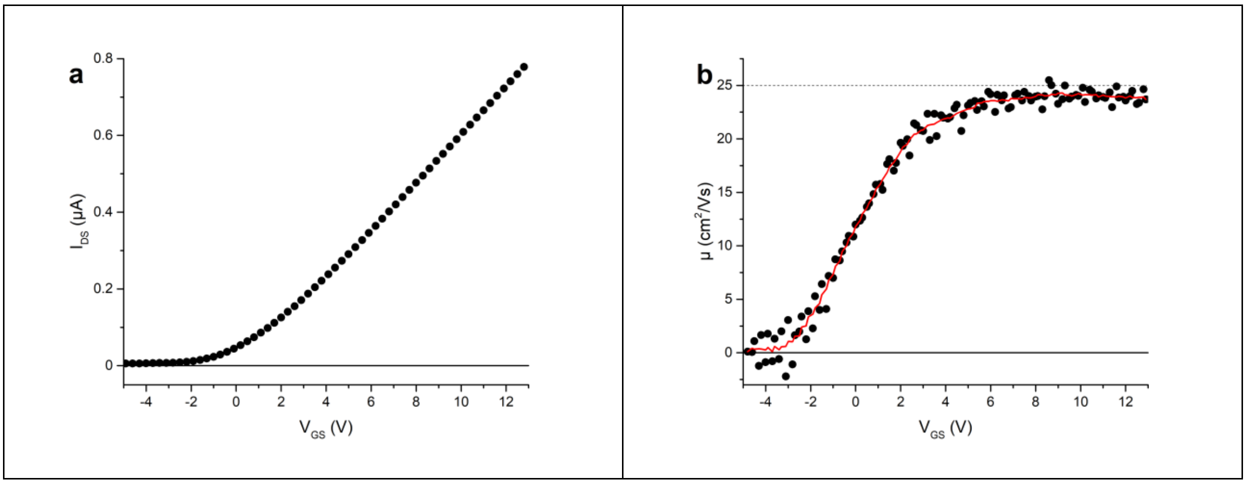

Figure S1. Measured transfer curve (left) at V_{DS} = 0.5 V and corresponding linear mobility (right), the red line being a smoothening of the calculated $\mu_{lin.}$ vs V_{GS} where the latter has been corrected for the threshold voltage. We used $\mu_{lin.} = \frac{dI_{DS}}{dV_{GS}} \frac{L}{W} \frac{1}{V_{DS}} \frac{1}{C_i}$ with a measured value of C_i = 21 nF/cm^2 and L = 231 μm and W = 60 μm to derive the mobility, which is valid at $V_{GS} - V_{th} \gg V_{DS}$, where the transistor operates in the linear region.

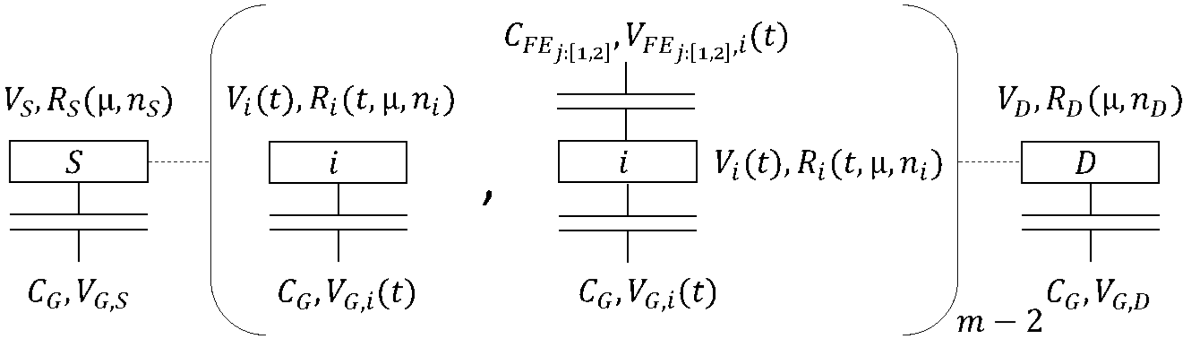

Figure S2. Illustration of the four different elements used to describe the grid cells in the 1D drift-only model accompanied by relevant parameters included in the simulations. The grid cells are capacitively coupled to either only the gate or to both the gate and a finger electrode from set j:[1, 2], determined by the position of the grid cell. The thickness of the etch stop layer between the IGZO channel and the finger electrodes is half that of the dielectric layer between the gate and the channel which is expressed by a doubling of the capacitance compared to the gate. In simulations for our device A = 4 μm, B = 16 μm, with 16 repeat units (as defined in Figure 1) plus one extra finger electrode for set 1 and 5 μm horizontal distance from source and drain to closest finger electrode. We use a cell size of 500 nm and thus 794 grid cells (first and last grid cells are the source and drain contact, respectively, while the others make up the channel). Nominal geometric capacitance values of C_G=17 nF/cm^2 and $C_{FE_{j:[1,2]}}$=34 nF/cm^2 for SiO_2 are used to enable direct comparison with 2D drift-diffusion simulations, see also Figures S3 and S6. The source and drain capacitances C_S and C_D are set equal to C_G; the density at the source and drain is fixed such that the source and drain potentials have the desired values according to $V_{S/D} = n_{S/D}/C_{S/D}$, defining the boundary conditions for the simulation.

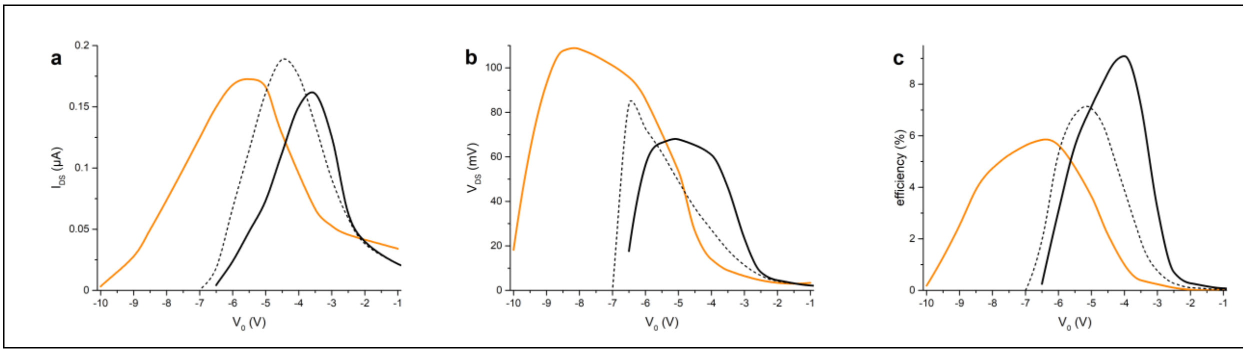

Figure S3. Time-averaged (DC) current I_{DS} (a), voltage V_{DS} (b), and efficiency, η, (c) at maximum power point for the 2D drift-diffusion model (orange symbols) and the 1D drift-only model (black symbols), for a single repeat unit of the base device with periodic boundary conditions (no contacts) at 5 MHz. The solid black line corresponds to 1D drift-only simulations for the full device with contacts (in (b) the voltage is scaled to the length of one repeat unit). The 2D drift-diffusion model is described in Ref. [1].

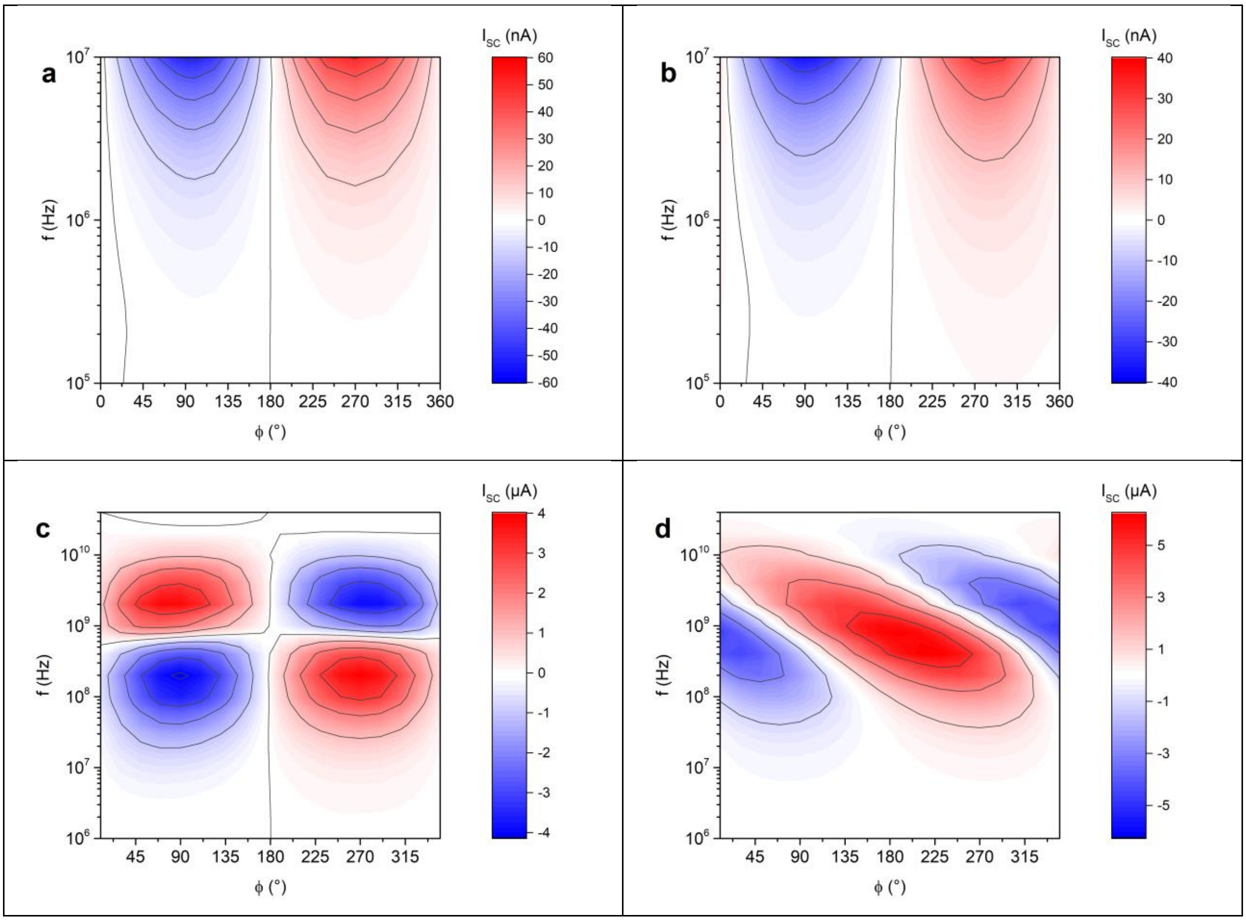

Figure S4. Measured (top) and simulated (bottom) short circuit device current I_{SC} versus phase difference and frequency for symmetric drive (left) and forward drive (right) for device*: A* = 2 μm, *B* = 8 μm, and the horizontal distance from the source and drain contacts to the closest finger electrode is 2.5 μm (all else being the same as the base device). Measurements carried out at V_0 = -2 V and V_0 = 0 for symmetric and forward drive, respectively, and simulations at V_0 = -4 V. In the frequency range that can be supplied by the function generator the optimal ϕ with respect to I_{SC} (and output power) for both symmetric and forward drive are 90° and 270° (a, b). At (simulated) higher frequencies (c, d),

forward drive has a clear frequency dependence of the phase angle that gives the highest I_{SC} (and output power). Symmetric drive on the other hand yields the highest I_{SC} (and output power) at ϕ = 90° and ϕ = 270° over all frequencies, with a current reversal occurring at 0.7 GHz in simulations.

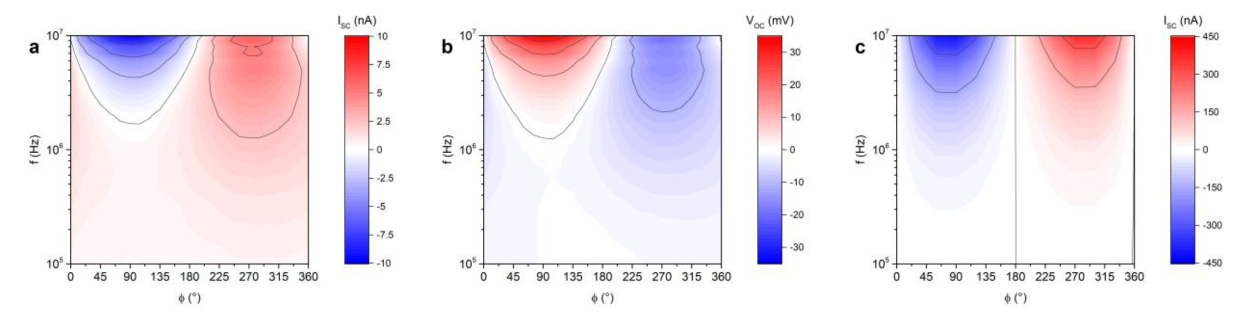

Figure S5. Measured short circuit current I_{SC} (a) and open circuit voltage V_{OC} (b) and simulated I_{SC} (c) versus phase difference and frequency. V_0 = -2.5 V for measurements and V_0 = -5 V for simulations. Both measurements and simulations yield maximum I_{SC} and maximum V_{OC} (and consequently maximum output power) close to ϕ = 90° and ϕ = 270° while minima are located close to 0° and 180°.

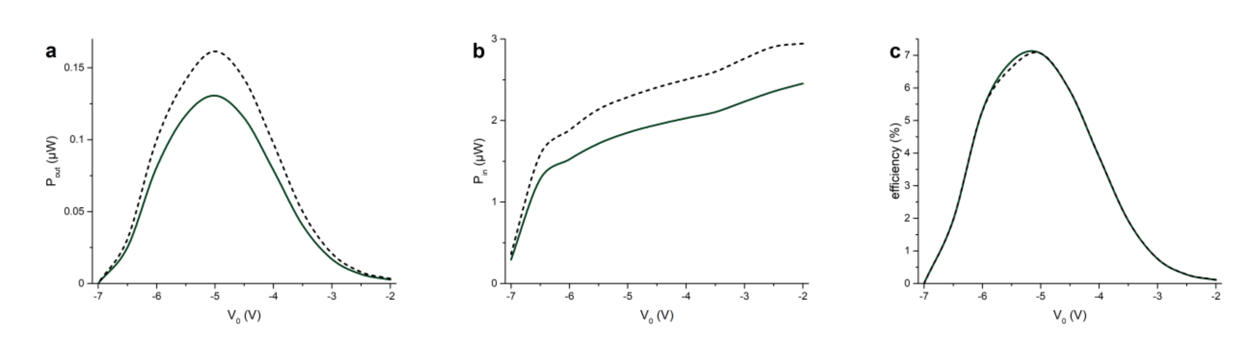

Figure S6. Evaluation of the sensitivity of the 1D drift-only simulations to the used capacitances. The dashed and solid lines indicate simulations with the measured (C_G=21 nF/cm^2) and nominal geometric (C_G=17 nF/cm^2) gate capacitance, respectively. The input and output power scale roughly linearly whereas their ratio, the efficiency, stays practically constant.

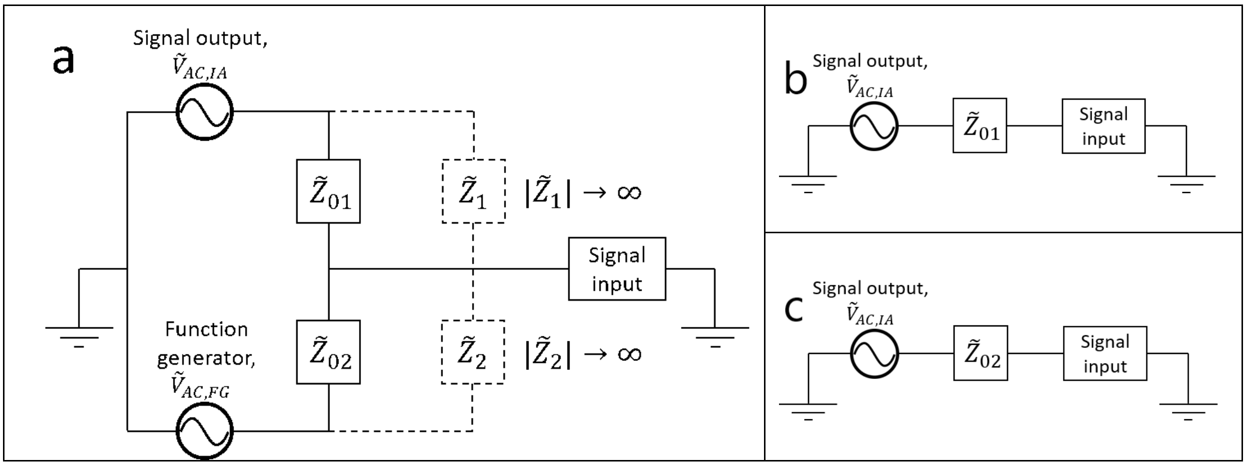

Figure S7. (a) Circuit diagram of the full device under depletion. Experimentally, $V_{GS} - V_{th}$ = -14 V and $V_0 = \frac{V_{GS}-V_{th}}{2}$. Under depletion there are no charge carriers in the semi-conducting channel, thus $|\tilde{Z}_1|$ and $|\tilde{Z}_2|$ become very large and can be disregarded. $\tilde{Z}_{01}$ and $\tilde{Z}_{02}$ can be determined sequentially by grounding one set of finger electrodes and connecting the signal output of the impedance analyzer to the other, resulting in the circuit shown in (b) when finger electrode set 2 is grounded and (c) when finger electrode set 1 is grounded.

The power to the device is supplied via the two sets of finger electrodes as they are connected to AC voltage sources. The input power of the ratchet can be calculated if we know the complex impedance of the individual sets of finger electrodes to the channel since we know the applied AC voltage and can express the related complex currents via these quantities. The components that dissipate power, being the background impedance $\tilde{Z}_{0,j}$ containing stray capacitances and cabling and the impedance of the finger electrodes set j $\tilde{Z}_j$, are modelled in the circuit diagram in **Figure S7**(a), with the addition that $|\tilde{Z}_1|$, $|\tilde{Z}_2|\rightarrow\infty$ during the special case of depletion. The complex impedance of the individual sets of finger electrodes to the channel, $\tilde{Z}_1$ and $\tilde{Z}_2$, cannot be measured directly. However, the stray capacitances and cabling, responsible for background dissipation, $\tilde{Z}_{01}$ and $\tilde{Z}_{02}$, can be measured sequentially by depleting the channel ($|\tilde{Z}_1|$, $|\tilde{Z}_2|\rightarrow\infty$) and grounding one set of finger electrodes (Figure S7(b,c)).

The complex impedance that is measured when driving the system in accumulation is, as described previously in Figure 5(a) of the main text:

$$\tilde{Z}' = |\tilde{Z}'| \cdot e^{i\theta'} = 1/\left(\left(\frac{\tilde{Z}_{01}+\tilde{Z}_1}{\tilde{Z}_{01}\tilde{Z}_1}\right) + \left(\frac{\tilde{Z}_{02}+\tilde{Z}_2}{\tilde{Z}_{02}\tilde{Z}_2}\right) \cdot \frac{1}{e^{-i\phi}}\right) \qquad \text{S1}$$

To determine the power dissipated via the finger electrodes we need to determine $\tilde{Z}_1 = |\tilde{Z}_1| \cdot e^{i\theta_1}$ and $\tilde{Z}_1 = |\tilde{Z}_1| \cdot e^{i\theta_1}$. We approximate $|\tilde{Z}_2|$ in terms of $|\tilde{Z}_1|$ as $|\tilde{Z}_2|=\frac{17}{16}\cdot|\tilde{Z}_1|$ via the relation $\frac{|\tilde{Z}_1|}{|\tilde{Z}_2|} = \frac{\#\ of\ set\ 1\ finger\ electrodes}{\#\ of\ set\ 2\ finger\ electrodes} = \frac{16}{17}$ due to the capacitive nature of the coupling of the finger electrodes to the channel. We have validated this assumption to be reasonable via our 2D drift-diffusion program. Accordingly, we need to determine $|\tilde{Z}_1|$, θ_1, and θ_2, which we do by fitting these so that the absolute impedance and argument of the RHS of Eq. S1**Error! Reference source not found.** correspond to the measured $|\tilde{Z}'|$ and θ', i.e. minimizing the sum of the squared errors (SSE) of the absolute impedance and the squared error of the argument. This is an underdetermined system, so we sweep the value of $|\tilde{Z}_1|$ while θ_1 and θ_2 are fitted for each step. Starting values of θ_1 and θ_2 need to be rationally selected to obtain a global minimum of SSE over the swept $|\tilde{Z}_1|$ and continuous parameters over offset voltage, as shown in **Figure S8**(a), black symbols. If the starting values for θ_1

and θ_2 are improper (red symbols) one can still obtain low SSE but one will not acquire an obvious minimum and the fitted values of θ_1 and θ_2 may vary greatly with $|\tilde{Z}_1|$ and between adjacent values of V_0.

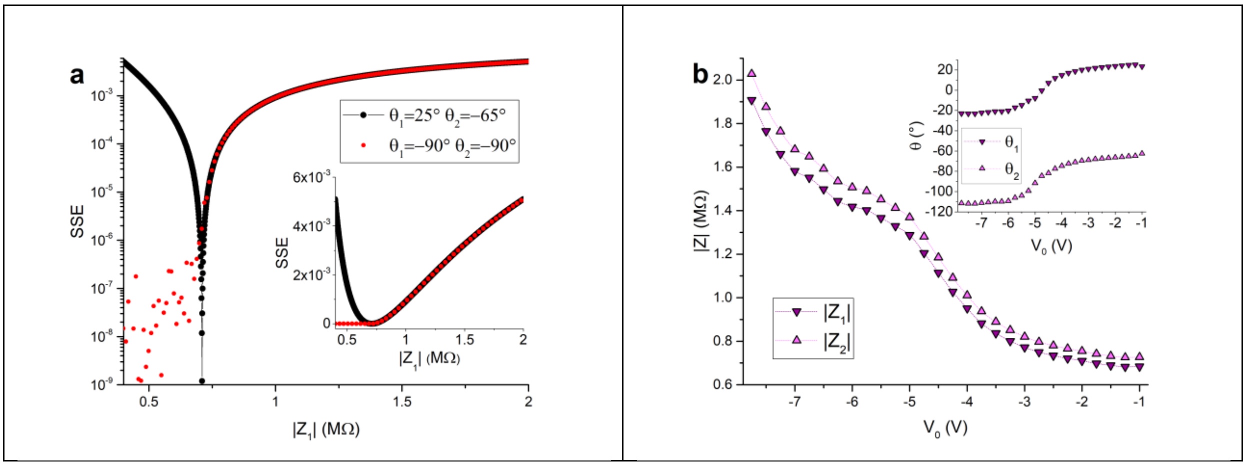

Figure S8. (a) Sum of squared errors of the absolute impedance and the argument between the LHS (obtained from measurements) and RHS (fitting θ_1 and θ_2 while sweeping $|\tilde{Z}_1|$, and $|\tilde{Z}_2|=\frac{17}{16}\cdot|\tilde{Z}_1|$) of Eq. S1 for offset -2 V and starting values θ_1 = 25° and θ_2 = -65° (black) and θ_1 = -90° and θ_2 = -90° (red). The inset shows the same data on a linear scale. (b) Absolute impedances $|\tilde{Z}_1|$ (dark magenta) and $|\tilde{Z}_2|$ (light magenta) versus offset obtained from points at minimum SSE with proper starting values of θ_1 = 25° and θ_2 = -65°. The inset shows the fitted arguments θ_1 (dark magenta) and θ_2 (light magenta) associated to these points.

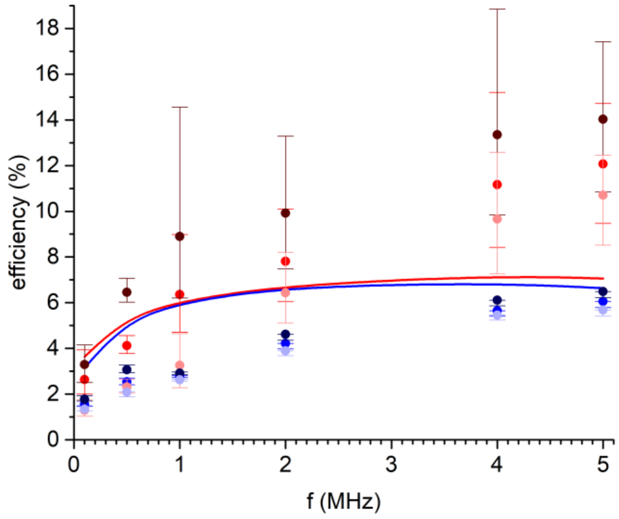

Figure S9. Influence of uncertainty in threshold voltage V_{th} on the efficiency η for each measured frequency. Measurements (symbols with error bars) and simulations (full lines) for ϕ = 270° (red) and ϕ = 90° (blue), where darker and lighter color correspond to, respectively, +0.25 V and -0.25 V shifts of V_{th} during the measurement of P_{in} relative to the value during the measurement of P_{out}. Symbols in measurements correspond to $P_{in} = \frac{P_{in,SC}+P_{in,OC}}{2}$, and the upper and lower error bar correspond to P_{in} weighted with respect to voltage at maximum power point, V_{MPP},: $P_{in,SC} - \frac{V_{MPP}}{V_{SC}}(P_{in,SC} - P_{in,OC})$

and P_{in} weighted with respect to current at maximum power point, I_{MPP},: $P_{in,OC} + \frac{I_{MPP}}{I_{SC}}(P_{in,SC} - P_{in,OC})$, where MPP is the maximum power point.

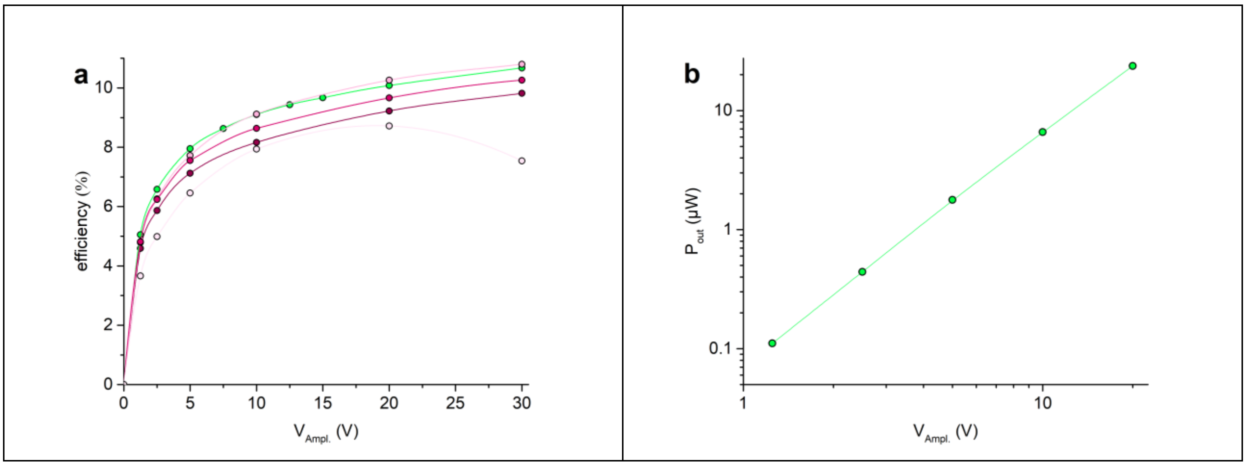

Figure S10. (a) Simulated efficiency, η, plotted versus oscillation amplitude, $V_{Ampl.}$, at 10 MHz for different values of V_{GS}: 2.5, 5, 10, 15, 20 V (light to dark red color, and green V_{GS} = 10 V the value used throughout the paper). There is a strong dependency on $V_{Ampl.}$ at lower amplitudes, but there are only small differences between chosen V_{GS} where V_{GS} = 10 V is optimal in the practical parameter range. (b) Simulated maximum output power, P_{out}, versus $V_{Ampl.}$ at 10 MHz. Both I_{DS} and V_{DS} at maximum power point, at the corresponding voltage offset that gives maximum output power, increase linearly with amplitude, and hence P_{out} increases quadratically, illustrated in the log-log plot (b). The dissipated power on the other hand increases sub-quadratically leading to the efficiency increase with increasing amplitude for the investigated range seen in (a).

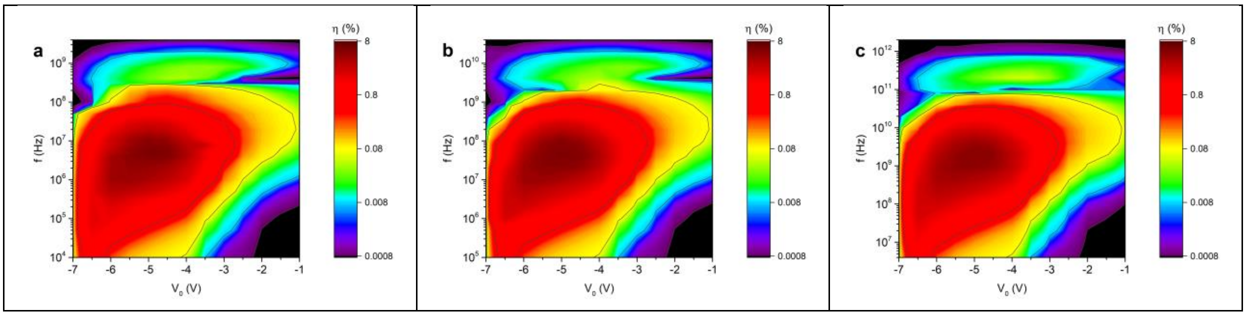

Figure S11. Simulated efficiencies over frequency for (a) base parameters, (b) ten times higher mobility: μ = 250 cm/Vs, and (c) decreased length scales by a factor 20: *A* = 0.2 µm*, B* = 0.8 µm, horizontal distance from source drain contacts to closest finger electrode is 0.25 µm, and finger electrode width is 0.1 µm. The relevant insight is that the magnitude of the efficiency maximum is unaltered despite the parameter change from (a) to (b) and from (a) to (c), however mobility and dimensionality shift the spectrum in linearly, respectively quadratically, in frequency space.

Supplementary reference

[1] E. M. Roeling, W. Chr. Germs, B. Smalbrugge, E. J. Geluk, T. de Vries, R. A. J. Janssen, M. Kemerink, *Nature Materials* **2011**, *10*, 51.
